# Decoronation as a Therapeutic Alternative for Ankylosis in Children and Adolescents for Vertical Bone Preservation and Growth: A Systematic Review

**DOI:** 10.3390/jcm14061945

**Published:** 2025-03-13

**Authors:** Aroa Bautista, James Ghilotti, Jose Luis Sanz, Carmen Llena

**Affiliations:** Department of Stomatology, Faculty of Medicine and Dentistry, Universitat de València, Gascó Oliag 1, 46010 Valencia, Spain; aroa.bautista@gmail.com (A.B.); jose.l.sanz@uv.es (J.L.S.); llena@uv.es (C.L.)

**Keywords:** decoronation, ankylosis, adolescent, young children, systematic review

## Abstract

**Background/Objectives**: Dentoalveolar ankylosis in adolescents involves a series of difficulties related to bone growth and development, especially in a vertical manner. A systematic review of studies on ankylosis in young permanent teeth treated by decoronation which considered the preservation/vertical growth of the alveolar bone as their main objective was carried out. **Methods**: The PRISMA 2020 guidelines were followed. Our research question was formulated using the PICO structure. Clinical cases or a series of cases of ankylosis in which a tooth had been treated with decoronation, with a minimum follow-up of one year, were included. The search was carried out in five databases. The selection of search terms was based on previous works within this framework and their most cited descriptors. The article selection and data extraction were carried out by two investigators. The JBI critical checklist of clinical cases was used for quality assessment. **Results**: Twelve articles were selected with a total of 23 cases that met the inclusion criteria. The mean age at the time of trauma was 9 years and the mean age at decoronation was 12.5 years. The traumatic event was avulsion in 10 out of the 14 cases. The upper right central incisor represented 62.5% of the sample compared to 25% for the contralateral tooth. The follow-up period ranged from 1 to 10 years. Vertical bone augmentation was found in all cases except in three cases where it remained stable. **Conclusions**: Based on the results of the present review, it can be concluded that decoronation is an effective technique for maintaining/stimulating vertical bone growth in young ankylosed permanent teeth and that complete root resorption is an important factor associated with vertical bone growth.

## 1. Introduction

Trauma-induced ankylosis is closely related to the progression of replacement root resorption. It is characterized by a loss of tooth tissue (cementum, dentin, and periodontal ligament (PDL)) with the subsequent replacement by osseous tissue, which results in the fusion of the root to the surrounding bone [1]. This type of resorption occurs following severe tooth trauma, such as intrusion, lateral luxation, or avulsion, where the PDL and a large portion of the root surface have been damaged [2]. The initial process is known as ankylosis, which is defined as a loss of the PDL. Ankylosis is effectively a fusion of the alveolar bone and dentin. Once ankylosis occurs, replacement resorption will follow as tooth tissues are resorbed by clastic cells and replaced by osseous tissue [3].

The pathogenesis of ankylosis of traumatic origin centers on injury to the periodontal ligament fibers. The necrosis of the periodontal ligament cells halts the normal mechanisms by which fibroblasts inhibit osteogenesis within the periodontium. This inhibition occurs through the release of local regulatory factors, such as cytokines and growth factors, which maintain the space between the root and the alveolar bone. Ankylosis is established not only through the inflammatory pathway and mechanical alterations in the periodontal ligament but also because very few cells survive, rendering them insufficient to suppress osteogenic activity. This alteration allows bone growth through the periodontal ligament, leading to the fusion of the alveolar bone and the root surface, ultimately resulting in the disappearance of the periodontal ligament [4].

Although novel diagnostic methods based on resonance frequency analysis (RFA), which quantifies the rigidity of the dental–bone connection, or the Periotest have been proposed, these methods have limited clinical application. RFA to assess the degree of stability of ankylosed teeth is expensive, has low availability in routine clinical practice, and is still an experimental test with low sensitivity. The Periotest did not exceed the accuracy of clinical examination for the diagnosis of ankylosis in post-trauma replanted permanent teeth and its firm tapping action is not well accepted by younger or more sensitive patients [5,6].

The percussion test, tooth mobility assessment, and the detection of eruption arrest remain the most commonly used diagnostic methods for ankylosis. Percussion elicits a characteristic metallic sound. Radiographic imaging confirms ankylosis when at least 20% of the root is affected [7]. Alamadi et al. compared periapical radiography, orthopantomography, and cone beam computed tomography (CBCT) for diagnosing ankylosis, reporting that CBCT is superior to conventional radiographs, especially in early stages [8].

Ankylosis in growing patients progresses rapidly. The affected tooth ceases to erupt, and its root resorbs, leaving it in an infraposition compared to the adjacent unaffected teeth. This discrepancy causes complications such as the tilting of adjacent teeth, the loss of arch length, and the localized arrest of alveolar ridge growth. The International Association of Dental Traumatology (IADT) recommends decoronation as a treatment technique for ankylosed incisors in growing patients to minimize the consequences of infraocclusion [9].

Several treatment options have been proposed for ankylosed teeth in growing children, including surgical luxation, orthodontic distraction, autotransplantation, or extraction followed by implant placement [10,11]. However, most of these techniques have been associated with an uncertain prognosis. In 1984, Malmgren introduced a more conservative treatment option known as decoronation. This procedure involves removing the crown of the ankylosed tooth below the cementoenamel junction and instrumenting the pulp canal to remove its contents while stimulating bleeding in the periapical area. The root is left in the socket to undergo resorption and be replaced by bone. Finally, the area is covered with a periosteal flap. This technique preserves the buccolingual width of the alveolar ridge and promotes vertical bone growth, thereby facilitating long-term prosthetic rehabilitation [12,13].

To our knowledge, no review has been conducted since Mohadeb et al.’s 2016 study on the efficacy of decoronation in preserving the alveolar bone after ankylosis [14]. Therefore, in the present work, we propose conducting a systematic review of clinical cases or case series examining the preservation and vertical growth of the alveolar bone following the decoronation of young permanent teeth, with a minimum follow-up period of one year.

## 2. Materials and Methods

The present study was conducted following the guidelines of the PRISMA (Preferred Reporting Items for Systematic Reviews) 2020 statement [15]. It was registered in PROSPERO under the following reference: [CRD42024625346].

### 2.1. Study Question and Eligibility Criteria

Studies presenting clinical cases or case series of ankylosis in young permanent teeth were considered for inclusion. Eligibility criteria were established according to the PICO model, as follows:

- Population (P): Young permanent teeth with ankylosis.
- Intervention (I): Decoronation of teeth.
- Comparison/control (C): Not applicable, since the objective is to review clinical cases or case series.
- Outcome (O): Maintenance/growth of the vertical supporting bone.

### 2.2. Inclusion and Exclusion Criteria

Studies were included if they presented clinical cases or case series of ankylosis in young permanent teeth, with at least one case treated with decoronation, a minimum follow-up of one year, and an evaluation of bone maintenance or vertical growth over time. Studies were excluded if they described cases of dental ankylosis in adult permanent teeth, cases where orthodontic treatment or surgery was performed to reposition the tooth, or cases where the decoronation procedure involved bone regeneration with xenografting. In vitro studies, the literature reviews, or experimental studies in animal models were also excluded.

### 2.3. Data Sources and Search Strategy

An electronic search was performed in the following databases: Medline, Scopus, Web Of Science, Embase, and Lilacs, with individual advanced search strings using the following keywords: “ankyl*”, “ankylosis”, “infraocclusion”, “infraposition”, “Tooth”, “teeth”, “root”, “dent*”, “alveol*”, “dentoalveolar”, “trauma*”, “avuls*”, “replacement resorption”, “resor*”, “luxa*”, “young”, “child*”, “decorona*”, “decoronation”, and “ridge preservation”, combined using the Boolean operators “AND” and “OR”. The search was last updated on 13 December 2024.

The selection of search terms was based on previous studies within this framework and their most cited descriptors. In addition, references of the included studies were manually screened after the selection process to verify additional potentially eligible studies. The advanced search strings and findings for the independent and combined search fields are presented in Table 1.

### 2.4. Study Screening and Selection Process

After searching each database, records were imported to the Mendeley reference management software (V.1.19.8) (Elsevier, AMS, Amsterdam, The Netherlands), and duplicate records were discarded. The resulting articles’ titles and abstracts were screened by two researchers. In the case of discrepancies, the two researchers discussed them and agreed on their inclusion or exclusion. In a second phase, the full texts were screened to decide which articles were finally included in the qualitative synthesis, after applying the inclusion and exclusion criteria. The concordance between the reviewers yielded a Kappa of 0.87.

### 2.5. Data Extraction and Recorded Variables2.6. Quality Assessment

A table was prepared containing the variables to be recorded for each study. The data collected were study characteristics, patient characteristics, methodology, and results. The authors and years of publication as well as the type of study were recorded as study char-acteristics. Among the patient characteristics, the following were collected: sex of the pa-tient, what type of trauma the tooth suffered and the age at which it occurred, time elapsed until ankylosis was diagnosed and in which tooth, age at which decoronation was per-formed, other treatments the patient may have received, and the degree of infraocclusion presented at the time of decoronation in three levels, depending on the distance between a line drawn along the mucogingival junction of the adjacent teeth and a line drawn along the mucogingival junction of the ankylosed tooth: A. Index 1. Minimal, <1/8 of the crown height. B. Index 2. Moderate, ≥1/8 and <1/4 of crown height. C. Index 3. Severe, ≥1/4 and <1/2 of crown height. D. Index 4. Extreme, ≥1/2 of the crown height. Methodological varia-bles included the follow-up period after decoronation to evaluate bone evolution and the type of method for such evaluation. Result variables included the final vertical bone out-come (loss/stabilization/gain). Data extraction was also performed by two authors (AB and CL).

### 2.6. Quality Assessment

The critical clinical case checklist provided by JBI, published in their manual “JBI REVIEWER’S MANUAL” in June 2020 [16], was used. This checklist is based on the CARE guidelines, which set out the standards for case reporting [17]. Two investigators performed the quality analysis. The interrater agreement was 0.89.

## 3. Results

### 3.1. Search Results and Study Selection

**Figure 1:**
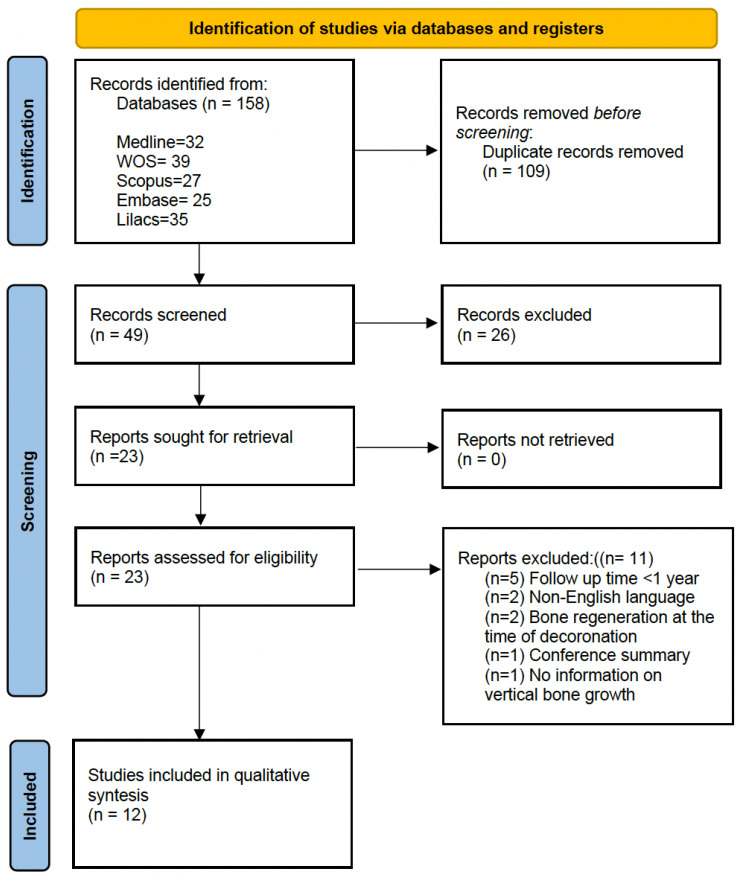
Flowchart of studies included in this study (PRISMA 2020, Page et al., 2021) [14].

The results of the electronic database search and study selection process were performed following the PRISMA 2020 guidelines [15] and are shown in the flowchart in Figure 1.

Searches in individual databases identified a total of 158 records: (Medline: 32, Scopus: 27, Embase: 25, Web of Science: 39, and Lilacs: 35). Using the ‘check for duplicates’ tool of the reference management software, duplicate records were discarded (n = 109). From the resulting 49 records, 26 were excluded after assessing compliance with the eligibility criteria by examining titles and abstracts. Twenty-three articles remained for full-text evaluation. Finally, 12 articles were included in the qualitative analysis.

The studies finally included were Araujo et al., 2023 [18]; Calasans-Maia et al., 2014 [19]; Diaz et al., 2007 [20]; Diniz et al., 2015 [21]; Han et al., 2024 [22]; Jaikaria et al., 2019 [23]; Lima et al., 2017 [24]; Malmgren et al., 2015 [25]; Steiner et al., 2020 [26]; Tsukiboshi et al., 2014 [27]; Walia et al., 2019 [28]; Zhang et al., 2021 [29]. One of the selected articles, that of Tsukiboshi et al., 2014 [27] was composed of a series of four cases, from which only one met the eligibility criteria.

Eleven articles were discarded after full-text evaluation. Five of them—Filippi et al., 2001 [30]; Gaspirc et al., 2022 [31]; Sala et al., 2017 [32]; Sapir et al., 2009 [33]; Siddiqui et al., 2016 [34]—did not provide follow-up longer than one year after decoronation. Lin et al., 2013 [35] evaluated the evolution of the bone in the buccal–palatal direction and not vertically. Madureira et al., 2022 [36] and Cohenca et al., 2006 [37] performed bone regeneration at the time of decoronation. Turjanski et al., 2022 [38] is part of an abstract of a congress book. The two remaining articles were discarded due to the language of their full text (Korean and German).

### 3.2. Result of the Quality Analysis

After applying the critical appraisal tool for clinical cases following the JBI REVIEWER’S MANUAL [16] guidelines (Table 2), from the twelve case reports and case series, six had a score of 8/8, five had a score of 7/8, and one had a score of 6/8, so eleven studies could be considered high quality (more than 85% of affirmative responses in the paper) and one moderate, confirming the reliability of the results.

### 3.3. Methodology of the Studies

The documentation of cases in the articles is relatively homogeneous. Many authors took intraoral photographs before decoronation, during the procedure, after the procedure, and in the follow-up visits (Araujo et al., 2023 [18]; Calasans-Maia et al., 2014 [19]; Diaz et al., 2007 [20]; Díniz et al., 2015 [21]; Han et al., 2024 [22]; Jaikaria et al., 2019 [23]; Lima et al., 2017 [24]; Steiner et al., 2020 [26]; Tsukiboshi et al., 2014 [27]; Walia et al., 2019 [28]). The performance of anterior photographs takes on great importance in these cases, as it allows the visualization of the degree of infraocclusion of the ankylosed tooth and the evolution of the gingival margin over time. However, in the studies in which case series were presented, images of only a few cases were provided as examples. Specifically, this is the case in the articles by Malmgren et al., 2015 [25] and Zhang et al., 2021 [29].

The authors also recorded patient demographics, relevant dental history to contextualize the ankylosis situation, and concomitant and/or subsequent treatments received by the patients in relation to decoronation.

For the evaluation of the bone level, the authors used periapical radiographs in all cases, in addition to CBCT in three of the articles (Calasans-Maia et al., 2014 [19]; Han et al., 2024 [22]; Tsukiboshi et al., 2014 [27]).

### 3.4. Results of the Studies

The results of the included studies are shown in Table 3. This analysis allows us to identify trends in demographic characteristics, treatment, and bone evolution of the alveolar ridge. The sum of the cases collected in the 12 articles provides a sample of 23 cases with decoronation treatments that met the inclusion criteria for this review.

#### 3.4.1. Characteristics of the Included Studies

The date of publication of the included studies ranged from 2007 to 2024, the most recent being by Han et al. in Korea [22]. Brazil and the United States are the countries with the highest number of articles, three (Calasans-Maia et al., 2014 [19]; Díniz et al., 2015 [21]; Lima et al., 2017 [24]) and two, respectively (Araujo et al., 2023 [18]; Steiner et al., 2020 [26]).

#### 3.4.2. Demographic Characteristics and Background

Regarding sex and age, most of the documented patients were men (74% men and 26% of women). In the articles presenting more than one case, this trend also coincides: Han et al., 2024 [22] with 66% men and 33% women, Zhang et al., 2021 [29] with 75% men and 25% women, and Malmgren et al., 2015 [25] with 74.67% men and 25.33% women, reflecting a higher prevalence of trauma in the male population.

The ages at the time of trauma vary between 6 and 17 years, the average being 9 years, coinciding with stages of incomplete root development in which the permanent teeth are more vulnerable.

Regarding the traumatic event, it was documented in 14 cases. Mainly avulsions were reported, specifically ten cases (Araujo et al., 2023 [18]; Diaz et al., 2007 [20]; Diniz et al., 2015 [21]; Han et al., 2024 [22]; Steiner et al., 2020 [26]; Tsukiboshi et al., 2014 [27]; Walia et al., 2019 [28]); two cases of intrusive dislocation (Calasans-Maia et al., 2014 [19]; Lima et al., 2017 [24]); three cases of lateral dislocation (Calasans-Maia et al., 2014 [19]); and one case of extrusive dislocation (Jaikaria et al., 2019 [23]). These results confirm that severe traumas are the main predisposing factors for ankylosis.

#### 3.4.3. Clinical Features and Treatment

All the studies present cases of ankylosis in permanent maxillary incisors in young patients. The right maxillary central incisor represents 62.5% of the sample with respect to 25% of its contralateral. The left and right maxillary lateral incisors represent 6.25% of the total sample, respectively.

Regarding the time to diagnosis of ankylosis, the intervals vary from diagnoses as early as one month after reimplantation (Steiner et al., 2020 [26]) to 3.5 years in the study by Han et al., 2024 [22]. In some studies, the elapsed time is not clearly specified (Araujo et al., 2023 [18]; Malmgren et al., 2015 [25]; Tsukiboshi et al., 2014 [27]; Zhang et al., 2021 [29]). The median time to diagnosis of ankylosis in the set of studies analyzed was 17 months.

The degree of infraocclusion of the reported cases varies from grades 1 to 3. In all cases, except in one of those presented by Han et al., 2024 [22], the degree of ankylosis could be extracted either from the author’s own description of the case or from the analysis of the attached photographs. To evaluate the degree of infraocclusion in the photographs, we followed what was described by Malmgren in his 2002 study. The case series of Malmgren et al., 2015 [25] and Zhang et al., 2021 [29], collect cases of infraocclusions of grades 1 to 3 and grades 1 and 2, respectively, without specifying how many cases correspond to each grade. From the remaining cases (n = 11), nine of them present a grade 1 infraocclusion (Araujo et al., 2023 [18]; Calasans-Maia et al., 2014 [19]; Díaz et al., 2007 [20]; Díniz et al., 2015 [21]; Han et al., 2024 [22]; Jaikaria et al., 2019 [23]; Steiner et al., 2020 [26]; Tsukiboshi et al., 2014 [27]; Walia et al., 2019 [28]), one case with a grade 3 infraocclusion (Lima et al., 2017 [24]), and only one with a grade 2 infraocclusion (Han et al., 2024 [22]).

Decoronation treatment was mainly performed in early adolescence (12.6 years on average in all the studies), which coincides with the peak of pubertal growth, which is established between 12.5 and 15 years in males and 10.5 to 13 years in females. Decoronation is often supplemented with orthodontics to manage occlusal problems or the displacement of adjacent teeth (Araujo et al., 2023 [18]; Calasans-Maia et al., 2014 [19]; Lima et al., 2017 [24]; Steiner et al., 2020 [26]; Walia et al., 2019 [28]). This combined approach prepares the area for future prosthetic rehabilitations by distributing the available space and correcting any malocclusions present.

#### 3.4.4. Bone Evolution

Follow-up periods after decoronation range from 1 year (Jaikaria et al., 2019 [23]; Tsukiboshi et al., 2014 [27]) to 10 years (Han et al., 2024 [22]). Studies with longer follow-ups report better bone outcomes, highlighting the importance of continuous monitoring.

Periapical radiographs are present in all cases to evaluate bone growth. In addition, three of the studies use CBCT to evaluate three-dimensional bone changes (Calasans-Maia et al., 2014 [19]; Han et al., 2024 [22]; Tsukiboshi et al., 2014 [27]).

The most common result in the cases studied is the development of vertical bone volume (Calasans-Maia et al., 2014 [19]; Díniz et al., 2015 [21]; Han et al., 2024 [22]; Lima et al., 2017 [24]; Malmgren et al., 2015 [25]; Steiner et al., 2020 [26]; Tsukiboshi et al., 2014 [27]; Walia et al., 2019 [28]; Zhang et al., 2021 [29]). Only in three cases did this increase not occur, although the preservation of the bone volume presented by the patient at the time of decoronation was achieved (Araujo et al., 2023 [18]; Diaz et al., 2007 [20]; Jaikaria et al., 2019 [23]).

The submerged portion of the incisor root acts as a matrix for bone apposition by substitution. Malmgren et al., 2015 [18] and Zhang et al., 2021 [29] do not provide data in this regard. In the remaining ten articles, eight cases were found in which complete root replacement by bone occurred (Díniz et al., 2015 [21]; Han et al., 2024 [22]; Lima et al., 2017 [24]; Tsukiboshi et al., 2014 [27]; Walia et al., 2019 [28]). In seven other cases, only a partial replacement of the root with bone was observed (Araujo et al., 2023 [18]; Calasans-Maia et al., 2014 [19]; Diaz et al., 2007 [20]; Jaikaria et al., 2019 [23]; Steiner et al., 2020 [26]).

## 4. Discussion

After a systematic search of the literature on ankylosis and decoronation as a therapeutic option, studies on the topic remain scarce, with most being reviews, case reports, or case series. The most extensive case series, as well as the most prolific author in terms of publications on decoronation, is Dr. Malmgren of Karolinska Institutet [25]. Malmgren described the decoronation technique as a conservative alternative for ankylosed permanent teeth, particularly in young patients [39]. Experimental studies conducted in the 1970s demonstrated that when vital tooth roots are submerged, minimal inflammatory changes occur. These findings formed the basis for developing the decoronation technique [30].

The primary objective of decoronation is to promote or at least preserve the volume of the bone crest and the vertical growth of the alveolar bone at the site of the ankylosed tooth [13]. This improves the prognosis for future prosthetic rehabilitation, although bone grafting may still be necessary if implants are planned [32].

To assess vertical growth, standardized long-cone periapical radiographs are sufficient. Malmgren et al. proposed a three-point assessment method to evaluate the vertical displacement of the alveolar bone level, using the cementoenamel junction of the homologous tooth as a reference. The levels are as follows: one = unchanged or reduced alveolar bone level, two = moderate increase in alveolar bone level, three = considerable increase in alveolar bone level [25].

This review focused on the vertical bone evolution following decoronation. In most of the included studies, vertical bone changes were assessed using periapical radiographs. Only three studies additionally provided CBCT images [19,22,27].

Some authors have incorporated simultaneous bone regeneration at the decoronation site, which can act as a confounding factor in assessing bone evolution. Due to this, two cases from the series presented by Tsukiboshi et al. [27] were excluded, along with cases reported by Madureira et al. [36] and Cohenca et al. [37].

In the decoronation technique, the decoronated root serves as a scaffold for new bone formation during root resorption. Initially, a new periosteum forms over the decoronated root, facilitating vertical alveolar growth. Subsequently, the interdental fibers severed during decoronation reorganize between adjacent teeth. The continued eruption of these teeth mediates marginal bone apposition through the dentoperiosteal fiber complex. Erupting teeth remain attached to the periosteum covering the alveolus and indirectly via the alveologingival fibers, which insert into the alveolar ridge and the lamina propria of the interdental papilla. Both structures generate tensile forces that promote bone apposition on the alveolar ridge. This theoretical biological explanation is based on known anatomical features, eruption processes, and clinical observations [40].

The more active osteogenic capacity in young patients enables faster bone regeneration due to higher metabolic activity compared to adults [39]. The density and vascularization of the alveolar bone facilitate root resorption. In this review, decoronation treatment was primarily performed in early adolescence (mean age: 12.6 years across all studies), aligning with the pubertal growth peak (12.5–15 years in males and 10.5–13 years in females). When decoronation is performed during this growth acceleration, bone apposition may be enhanced, along with the correction of localized maxillary growth deficiencies caused by ankylosis [25,29]. However, facial growth can also be influenced by genetic and epigenetic factors unique to each individual [41]. Root resorption typically occurs within 6 to 24 months [39]. Consequently, this review established a minimum follow-up period of 12 months for study inclusion. In contrast, a previous systematic review published in 2016 did not consider follow-up time as an eligibility criterion [13].

In this review, complete root replacement by bone was reported in eight cases [21,22,24,27,28], while seven cases exhibited partial root replacement [18,19,20,23,26]. In three of these cases, vertical bone was preserved without additional vertical growth [18,20,23]. Notably, studies in which vertical bone growth was observed had follow-up periods of at least two years, whereas in two of the three cases showing only stabilization, follow-up times were shorter [18,23]. Among cases with complete root resorption, vertical apposition occurred in all but two [19,26], with most studies maintaining a minimum follow-up of two years. In the three cases where vertical bone preservation without growth was observed, root resorption was partial [18,20,23]. These findings suggest that complete root resorption favors vertical bone growth, with a follow-up period of at least two years being necessary for proper evaluation.

One limitation of this study is that, while the included studies have a low risk of bias according to the JBI scale for assessing bias in clinical case studies and case series, this type of study is inherently susceptible to bias. The retrospective nature of the data limits the investigator’s control over many relevant variables. Given that for this type of pathology it is not possible to propose studies with a high level of evidence such as clinical trials, it would be necessary to establish diagnostic protocols and standardized treatment indications in order to compare published cases or case series and provide the clinician with guidelines for their application.

## 5. Conclusions

The primary objective of decoronation is to promote or at least preserve the volume of the bone crest and the vertical growth of the alveolar bone at the site of the ankylosed tooth. This improves the prognosis for future prosthetic rehabilitation. Therefore, it should be considered a good therapeutic alternative in young ankylosed permanent teeth.

## Figures and Tables

**Table 1:** Search strategy and findings.

Database	Search String	Findings
**Medline**	#1: (Ankyl* OR ankylosis OR infraocclusion OR infraposition)	32,245
	#2: (Tooth OR teeth OR root OR dent* OR alveol* OR dentoalveolar)	1,553,421
	#3: (Trauma* OR avuls* OR replacement resorption OR resor* OR luxa*)	823,358
	#4: #1 AND #2 AND #3	1253
	#5: (Young OR child*)	4,697,124
	#6: (Decorona* OR decoronation OR “ridge preservation”)	1733
	**#4 AND #5 AND #6**	**32**
**Scopus**	#1 TITLE-ABS-KEY (Ankyl* OR ankylosis OR infraocclusion OR infraposition)	47,396
	#2 TITLE-ABS-KEY (Tooth OR teeth OR root OR dent* OR alveol* OR dentoalveolar)	2,129,074
	#3 TITLE-ABS-KEY (Trauma* OR avuls* OR replacement resorption OR resor* OR luxa*)	13,876
	#4: #1 AND #2 AND #3	484
	#5: (Young OR child*)	5,755,688
	#6: (Decorona* OR decoronation OR “ridge preservation”)	1984
	#4 AND #5 AND #6	**27**
**Embase**	#1 (Ankyl* OR ankylosis OR infraocclusion OR infraposition)	57,271
	#2 (Tooth OR teeth OR root OR dent* OR alveol* OR dentoalveolar)	1,867,027
	#3 (Trauma* OR avuls* OR replacement resorption OR resor* OR luxa*)	139,424
	#4: #1 AND #2 AND #3	760
	#5: (Young OR child*)	5,671,227
	#6: (Decorona* OR decoronation OR “ridge preservation”)	1470
	#4 AND #5 AND #6	**25**
**Web of Science**	#1: TS = (Ankyl* OR ankylosis OR infraocclusion OR infraposition)	56,813
	#2: TS = (Tooth OR teeth OR root OR dent* OR alveol* OR dentoalveolar)	3,418,092
	#3: TS = (Trauma* OR avuls* OR replacement resorption OR resor* OR luxa*)	1,242,532
	#4: #1 AND #2 AND #3	1685
	#5: TS = (Young OR child*)	7,316,598
	#6: TS = (Decorona* OR decoronation OR “ridge preservation”)	2650
	#4 AND #5 AND #6	**39**
**Lilacs**	#1 (Ankyl* OR ankylosis OR infraocclusion OR infraposition)	1199
	#2 (Tooth OR teeth OR root OR dent* OR alveol* OR dentoalveolar)	136,762
	#3 (Trauma* OR avuls* OR replacement resorption OR resor* OR luxa*)	58,284
	#4: #1 AND #2 AND #3	2164
	#5: (Young OR child*)	176,453
	#6: (Decorona* OR decoronation OR “ridge preservation”)	83
	#4 AND #5 AND #6	**35**

**Table 2:** Quality assessment of the included studies.

	Were the Patient’s Demographic Characteristics Clearly Described?	Was the Patient’s History Clearly Described and Presented in the Form of a Chronology?	Was the Patient’s Current Clinical Status at the Time of Presentation Clearly Described?	Were the Diagnostic Tests or Methods and Results Clearly Described?	Were the Intervention(s) or Treatment Procedure(s) Clearly Described?	Was the Post-Intervention Clinical Status Clearly Described?	Were Adverse Events (Harms) or Unforeseen Events Identified and Described?	Does the Clinical Case Provide Lessons for Implementation?	Score
Araújo et al., 2023 [18]	Unclear	Unclear	Yes	Yes	Yes	Yes	Yes	Yes	7
Calasans-Maia et al., 2014 [19]	Unclear	Yes	Yes	Unclear	Yes	Yes	Yes	Yes	6
Díaz et al., 2007 [20]	Yes	Yes	Yes	Yes	Yes	Yes	Yes	Yes	8
Díniz et al., 2007 [21]	Yes	Unclear	Yes	Yes	Yes	Yes	Yes	Yes	7
Han et al., 2024 [22]	Yes	Yes	Yes	Yes	Yes	Yes	Yes	Yes	8
Jaikaria et al., 2019 [23]	Yes	Yes	Yes	Yes	Yes	Yes	Yes	Yes	8
Lima et al., 2017 [24]	Yes	Yes	Yes	Yes	Yes	Yes	Yes	Yes	8
Malmgrem et al., 2015 [25]	Yes	Unclear	Yes	Yes	Yes	Yes	Yes	Yes	7
Steiner et al., 2020 [26]	Yes	Yes	Yes	Yes	Yes	Yes	Yes	Yes	8
Tsukiboshi et al., 2014 [27]	Yes	Yes	Yes	Yes	Unclear	Yes	Yes	Yes	7
Walia et al., 2019 [28]	Yes	Yes	Yes	Yes	Yes	Yes	Yes	Yes	8
Zhang et al., 2021 [29]	Yes	Unclear	Yes	Yes	Yes	Yes	Yes	Yes	7

**Table 3:** Study results.

Author and Year; Study Type	Gender; Age	Traumatic History; Tooth	Time Elapsed Until Diagnosis of Ankylosis	Degree of Underoclussion	Age at Decoronation	Follow-Up Time After Decoronation	Additional Treatments	Bone Maintenance Assessment Method	Bone Evolution
Araujo et al., 2023 [18] USA Case report	Male 9 years	Avulsion 1.1	NE	1	Not specified	19 months	Orthodontics	Periapical X-ray	Preservation of vertical volume; partial replacement of root with bone
Calasans-Maia et al., 2014 [19] Brazil Case report	Male 15 years	Intrusive luxation 1.2 Lateral luxation 1.1, 2.1	6 months	1	14 years	5 years	Orthodontics	Periapical X-ray CBCT	Vertical bone position; partial replacement of root with bone
Díaz et al., 2007 [20] Chile Case report	Male 8 years	Avulsion 1.1	3 months (percussion) 18 months (infraocclusion)	1	9.5 years	44 months	-	Periapical X-ray	Preservation of vertical volume; partial replacement of root with bone
Díniz et al., 2015 [21] Brazil Case report	Female 8 years	Avulsion 2.1	N/S	1	10 years	3 years	-	Periapical X-ray	Vertical bone position; complete replacement of root with bone
Han et al., 2024 [22] Korea Case series	Female 8 years and 8 months	Avulsion 1.1	2 months (metallic percussion, lost LP rx)	2	10 years and 3 months	4 years	-	Periapical X-ray	Vertical bone position; complete replacement of root with bone
	Male 8 years and 6 months	Avulsion 2.1, 2.2	4 months (Root resorption)	N/S N/S	13 years and 6 months	5 years	-	Periapical X-ray	Vertical bone position; complete replacement of root with bone
	Male 9 years and 3 months	Avulsion 1.1	3.5 years	1	12 years and 9 months	10 years	-	Periapical X-ray CBCT	Vertical bone position; complete replacement of root with bone
Jaikaria et al., 2019 [23] India Case report	Female 12 years	Lux. Extrusiva 2.1	2 years (metallic percussion)	1	14 years	1 year	-	Periapical X-ray	Preservation of vertical volume; partial replacement of root with bone
Lima et al., 2017 [24] Brazil Case report	Male 6 years	Lux. Intrusiva 1.1	6 months	3	10 years	5 years	Orthodontics	Periapical X-ray	Vertical bone position; complete replacement of root with bone
Malmgren et al., 2015 [25] Switzerland Case series	56 men 19 women 6, 8–17, 8 years 10, 7 ± 10, 4 years	N/S 1.1, 1.2, 2.1, 2.2	N/S	1, 2, 3	9, 3–22 years 14.9 ± 15 years	4.6 years (1–19.3 years)	-	Periapical X-ray	Considerable marginal bone development after decoronation
Steiner et al., 2020 [26] USA Case report	Male 9 years	Avulsion 1.1	1 month (metallic percussion)	1	14.5 years	2 years	Orthodontics	Periapical X-ray	Vertical bone position; hardly any replacement of root with bone
Tsukiboshi et al., 2014 [27] Japan Case report	Male 10 years	Avulsion 1.1	N/S	1	15.5 years	1 year	-	Periapical X-ray CBCT	Vertical bone position; complete replacement of root with bone
Walia et al., 2019 [28] Arab Emirates Case report	Female 8 years	Avulsion 1.1	6–9 months (metallic percussion and resorption observed in X-ray)	1	9.6 years	2.5 years	Orthodontics	Periapical X-ray	Vertical bone position; complete replacement of root with bone
Zhang et al., 2021 [29] China Case series	9 men 3 women 9, 35 ± 1, 23 years	N/S 1.1, 1.2, 2.1, 2.2	N/S	1, 2	13.1 ± 1.37 years	2–3 years	-	Periapical X-ray	Eleven of the twelve cases showed a considerable increase in bone levels

N/S: not specified.
